# Hypertrophic cardiomyopathy in infant newborns of diabetic mother: a heterogeneous condition, the importance of anamnesis, physical examination and follow-up

**DOI:** 10.1186/s13052-021-01145-x

**Published:** 2021-09-30

**Authors:** Alessia Claudia Codazzi, Rosario Ippolito, Cecilia Novara, Enrico Tondina, Rosa Maria Cerbo, Chryssoula Tzialla

**Affiliations:** 1 Pediatric Clinic, Fondazione IRCCS Policlinico San Matteo, Pavia, Italy; 2 Pediatric Clinic, Fondazione IRCCS Policlinico S. Matteo, University of Pavia, Pavia, Italy; 3 Neonatal Unit and Neonatal Intensive Care Unit, Fondazione IRCCS Policlinico San Matteo, Pavia, Italy

**Keywords:** Pompe disease, Interventricular septal hypertrophy, Fetal hyperinsulinemia

## Abstract

**Background:**

Hypertrophic cardiomyopathy (HCM) in neonates is a rare and heterogeneous disorder. HCM accounts for 25 to 40% of all pediatric cardiomyopathy cases and the highest incidence in pediatric population is reported in children < 1 year.

**Case presentation:**

we report two clinical cases of neonates, born to mothers respectively with a pre-pregnancy insulin-dependent diabetic mellitus type 2 and a suspected diabetes, with inadequate prenatal glycemic control for the first and underestimated glycemic control for the second case, with a different evolution.

In the first case, a slow evidence of improvement of the HCM was observed, persuading us to the diagnosis of a diabetes-related HCM; In the second case the progressive worsening of the HCM during follow-up in association with further investigations, resulted in the diagnosis of Pompe disease.

**Conclusions:**

Hypertrophic cardiomyopathy in newborns can be the clinical expression of different underlying disorders. We aim to show the importance both to reassess maternal and family history and critically evaluate the physical examination in order to address the correct differential diagnosis. Furthermore it is important to continue a regular cardiologic follow-up for this pathology with neonatal onset to prevent a poor prognosis.

## Introduction

Hypertrophic cardiomyopathy (HCM) in neonates is a rare and heterogeneous disorder characterized by histological and functional disruption of the myocardial structure/composition. HCM accounts for 25 to 40% of all pediatric cardiomyopathy cases and the highest incidence in pediatric population is reported in children < 1 year. There is a slight male predominance, and prevalence is higher in African American children than in white or Hispanic children [1] .

In the paediatric population HCM aetiology is mainly genetic/familial: about 50% of all paediatric cases are related to sarcomere protein mutations; about 30% is classified as follows: inborn errors of metabolism (glycogen storage diseases such as Pompe disease and Danon disease, disorders of fatty acid metabolism, lysosomal storage disorders such as mucopolysaccharidoses) and mitochondrial cardiomyopathies (respiratory chain complex deficiencies, mitochondrial syndromes), malformation syndromes associated with HCM (Costello syndrome, cardiofaciocutaneous syndrome and Noonan syndrome) and neuromuscular disorders, each contributing to about 10% of all cases [2]. According to the last evidence a definite genetic diagnosis can be reached in nearly 80% of patients with HCM of childhood onset [3].

Among infants, etiologic diagnosis of HCM is particularly challenging for clinicians: generally the highest incidence of inborn errors of metabolism and neuromuscular disorders is observed, and despite the advances in genetic and metabolic diagnosis, about 50% of HCM cases under one year of age remains idiopathic. Furthermore, survival in infants is much poorer than in older groups [1].

A particular aetiology specific of neonatal age is the development of HCM in Infants of Diabetic Mother (IDMs), with an incidence range from 13 to 44% [4] This condition is usually explained by Pederson maternal hyper-glycemia fetal hyperinsulinemia hypothesis: the anabolic effect of fetal hyperinsulinism related to maternal hyper-glycemia, which persists transiently in the neonatal period, leads to an increase in the synthesis and deposition of fat and glycogen in the myocardial fetal cells. The pattern of HCM related to hyperinsulinemic states is often described as septal hypertrophy or left ventricular outflow tract obstruction [4, 5]. In this condition, HCM is usually reversible, as the stimulus for the insulin production disappears, and in most situations, it is no longer detected on ultrasound after 6 months postnatally [5, 6].

We describe two cases of HCM in female neonates, from mothers with personal history of diabetes with a different evolution during the echocardiographic follow up. We aim to show the importance of detailed anamnesis, physical examination and a strict follow-up for this pathology with neonatal onset, that requiring a continue regular cardiologic monitoring to prevent a poor prognosis in the first year of life.

## Case presentation

### Case 1

E.A is a female infant born at term (37 weeks and 4 days of gestational age) daughter of not consanguineous parents, of African origins, without family history of cardiac disease, her mother was affected by pre-pregnancy poorly controlled insulin-dependent diabetic mellitus type 2. The maternal glycosylated hemoglobin at the diagnosis was 17% and because of the poor control of oral therapy she rapidly started insulin therapy. In the third trimester, her glycosylated hemoglobin was 7% with insulin therapy. Fetal echocardiography at 35th week of gestational age showed a severe HCM which progressively worsened during the following two weeks, a systolic anterior motion (SAM) of the mitral valve was present with left ventricular (LV) outflow tract obstruction; no hydrops fetalis was detected.

**Fig. 1:**
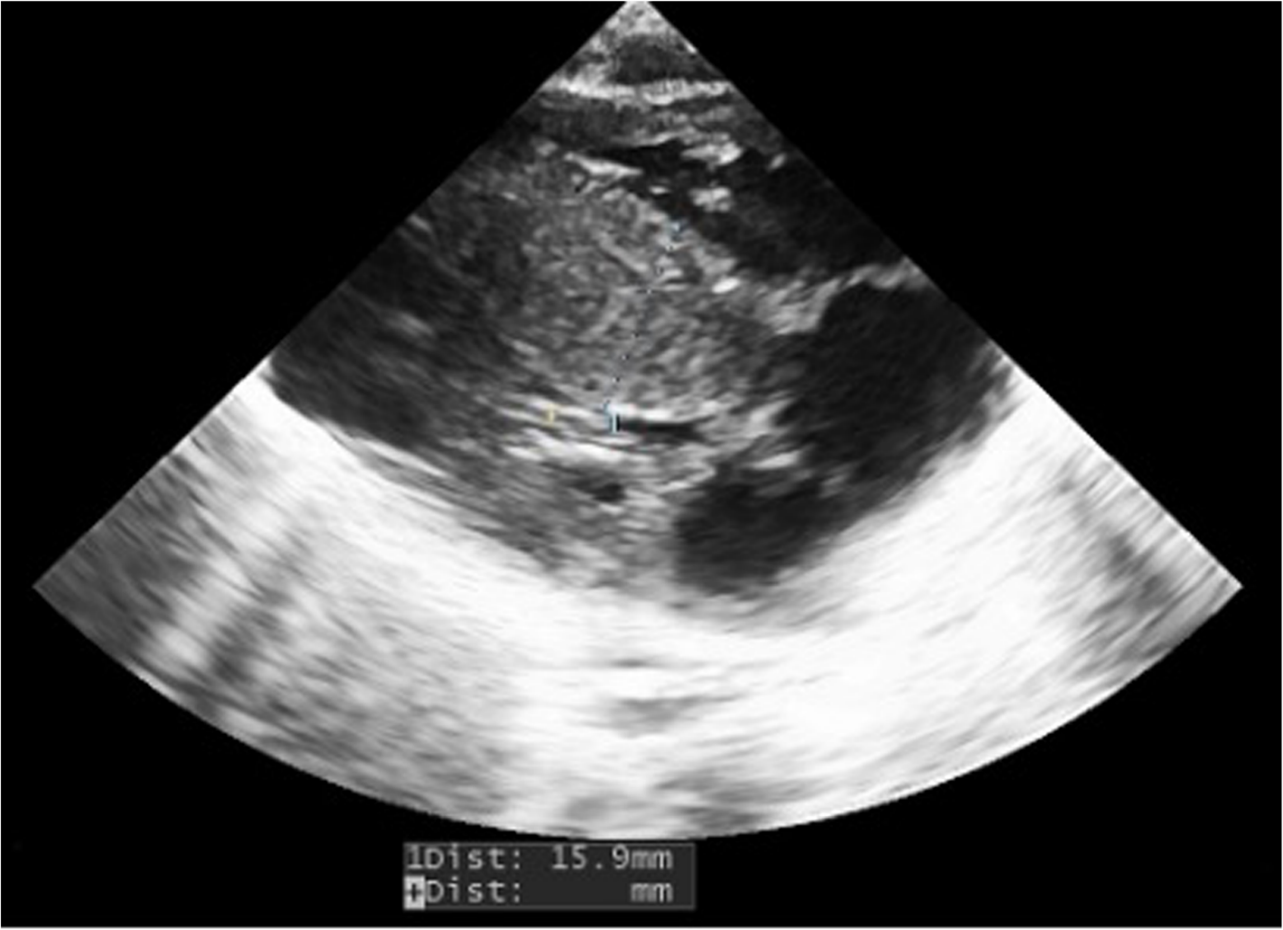
Echocardiogram performed after birth showed severe Left Ventricular (LV) hypertrophy and interventricular septal hypertrophy (IVSd of 15.9 mm, z-score 6.81) with a significant reduction in the ventricular cavity

**Fig. 2:**
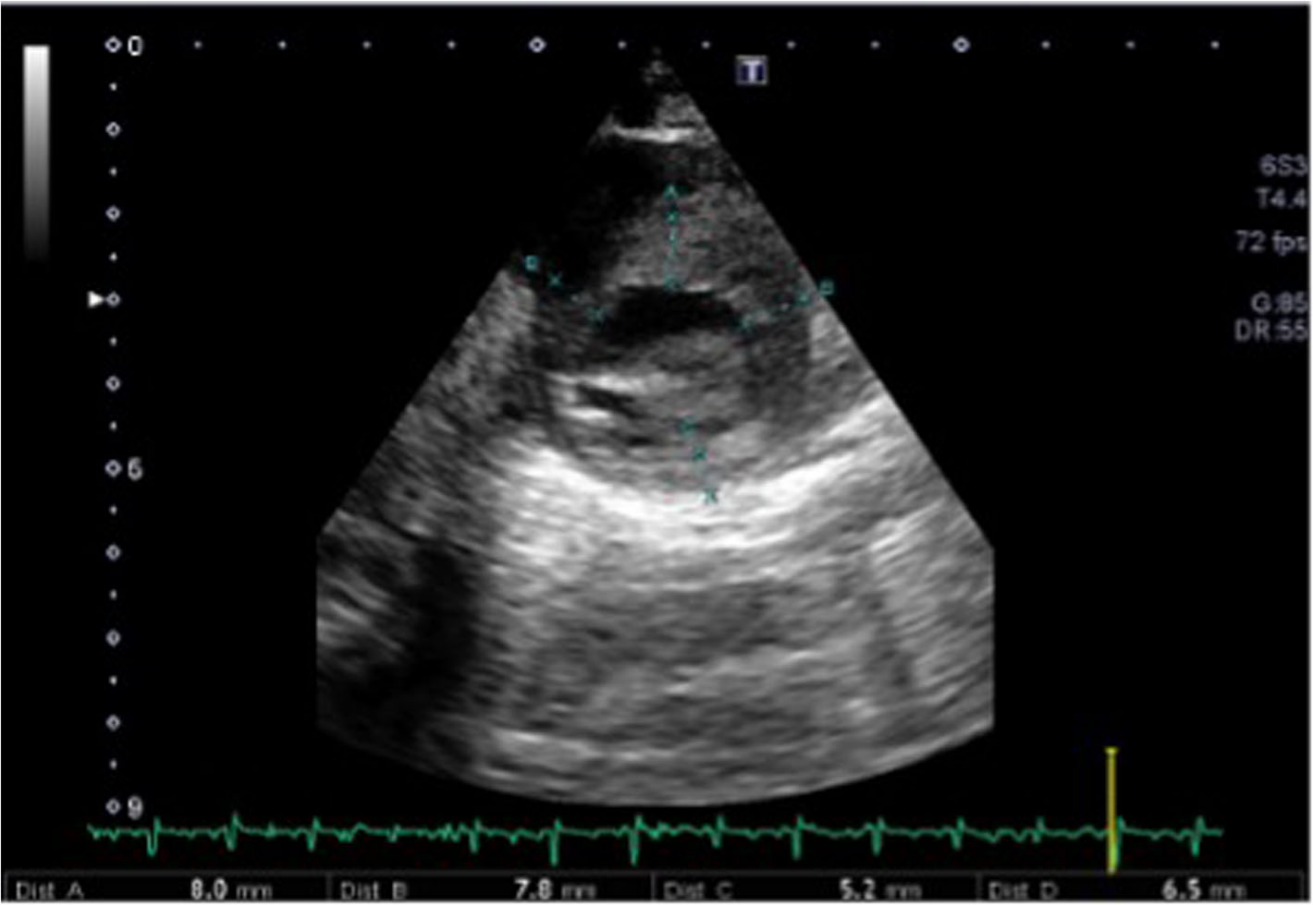
Echocardiogram performed (6 months of age) showed a significant reduction of the interventricular septal hypertrophy (IVSd of 8 mm; z-score 3.01)

Caesarean section was then performed on cardiologic indication, for evidence of rapid evolution of fetal myocardial hypertrophy. Birth weight was 3840 g (LGA), Apgar score of 1/6/7/8 at 1, 5, 10 and 20 min respectively. After neonatal resuscitation, she was transferred in Neonatal Intensive Care Unit (NICU). In the first day of life, hypoglycemia was detected and treated with intravenous (IV) glucose. Echocardiogram performed after birth showed severe Left Ventricular (LV) hypertrophy and interventricular septal hypertrophy (IVSd of 15.9 mm, z-score 6.81) with a significant reduction in the ventricular cavity, End Diastolic Volume (EDV of 2.4 ml) and LV outflow tract obstruction with a maximum intraventricular pressure gradient of 48.7 mmHg, and medium 12 mmHg (Fig. 1). The ECG findings (high QRS voltage in precordial leads) were confirming HCM. A therapy was immediately started with beta-blockers: I. V esmolol (starting dose 50 γ/kg/min increased to 100 γ/kg/min). After the improvement of the echocardiographic features the therapy was shifted to oral propranolol (3–4 mg/kg/day) well tolerated during follow up. In consideration of the severity of the HCM further investigations in order to exclude metabolic and genetical underlying conditions were performed: muscolar and cutaneus biopsy for mithocondrial diseases, enzyme blood tests for metabolic diseases, Next Generation Sequencing (NGS) for sarcomeric protein genes mutations were all negatives. During the cardiological follow-up (performed weekly for the first month, then every two weeks until the third month and then monthly) a slow evidence of improvement of the HCM was observed. The last echocardiogram performed (6 months of age) showed a significant reduction of the interventricular septal hypertrophy (IVSd of 8 mm; z-score 3.01), a normalization of the left ventricular cavity (EDV 11.4 ml) with decreased ecocardiographic signs of LV outflow tract obstruction (maximum intraventricular pressure gradient 14 mmHg, medium 6 mmHg) (Fig. 2).

The Holter ECG performed during follow-up confirmed the HCM, without evidence of arrhythmic events.

The child was always asymptomatic, with a regular growth by maternal breastfeeding.

Oral propranolol therapy is still ongoing at 1 mg/kg/day and she in on cardiologic follow-up every three months.

### Case 2

**Fig. 3:**
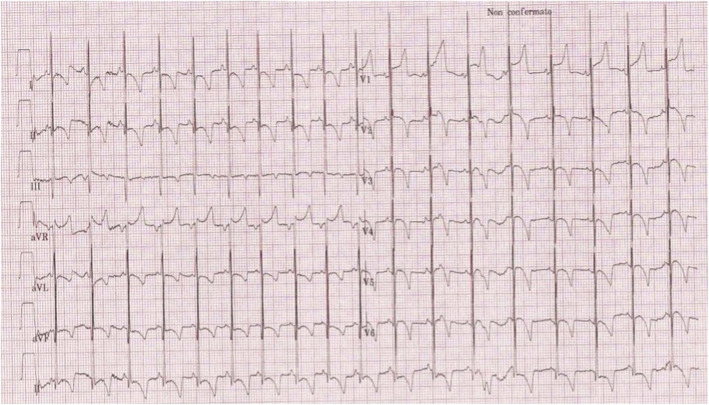
ECG: high voltage QRS complexes in precordial leads and a short PR interval

K. A. is female infant born at term (37 weeks and 3 days of gestational age) from a dichorionic diamniotic twin pregnancy, by caesarean section performed for unstoppable labour. The Apgar score was 9 and 10 at 1 and 5 min, respectively, and birth weight was 2275 g (Small for Gestational Age, SGA). The patient was the third child of consanguineous parents without family history of cardiac disease and the mother at the time of delivery did not bring documentation relating to the pregnancy in progress, although she reported in the medical history a previous poorly controlled insulin-dependent diabetic mellitus type 2 found in the previous pregnancy. Unfortunately the hasty birth and the lack of documentation of the current glycemic balance allowed us to diagnose suspected gestational diabetes. A heart murmur was detected during the first hours of life, therefore an ECG (high QRS voltage in precordial leads) (Fig. 3) and echocardiogram were performed, showing moderate biventricular hypertrophy, with interventricular septal hypertrophy (SIVd 6.6 mm; Z-score 5.1, LVPWd 4.6 mm Z-score 4.75), mild RV outflow tract pressure gradient (8.5 mmHg), LV cavity with EDV of 5.3 ml, without LV outflow tract obstruction. LV systolic function was preserved (FE 74%, FS 40%), whereas she presented mild LV diastolic disfunction (E/A 1.13, E/E’ 14). Muscle tone, posture and activity were still within limits normal for gestational age.

After discharge a weekly cardiological follow-up was performed: a rapid progressive worsening of the HCM was observed and therapy with oral propranolol was started. Nevertheless, the ultrasonographic finding was not improving and the general conditions of the patient were progressively deteriorating (sucking weakness, soft hypotonia, poor growth), therefore she was readmitted to the NICU. The ECG revealed the appearance of a short PR interval as a pre-excitation pattern. For these reasons investigations in order to evaluate related conditions, particularly inborn errors of metabolism, included: muscolar biopsy, suggestive for glycogenosis, and the dried blood spot (DBS) test for alfa1,4-glucosidase activity, demonstrating a low activity suggestive for Pompe disease (Type II Glycogenosis). The diagnosis was then confirmed through genetic analysis by mutations in the GAA gene.

**Fig. 4:**
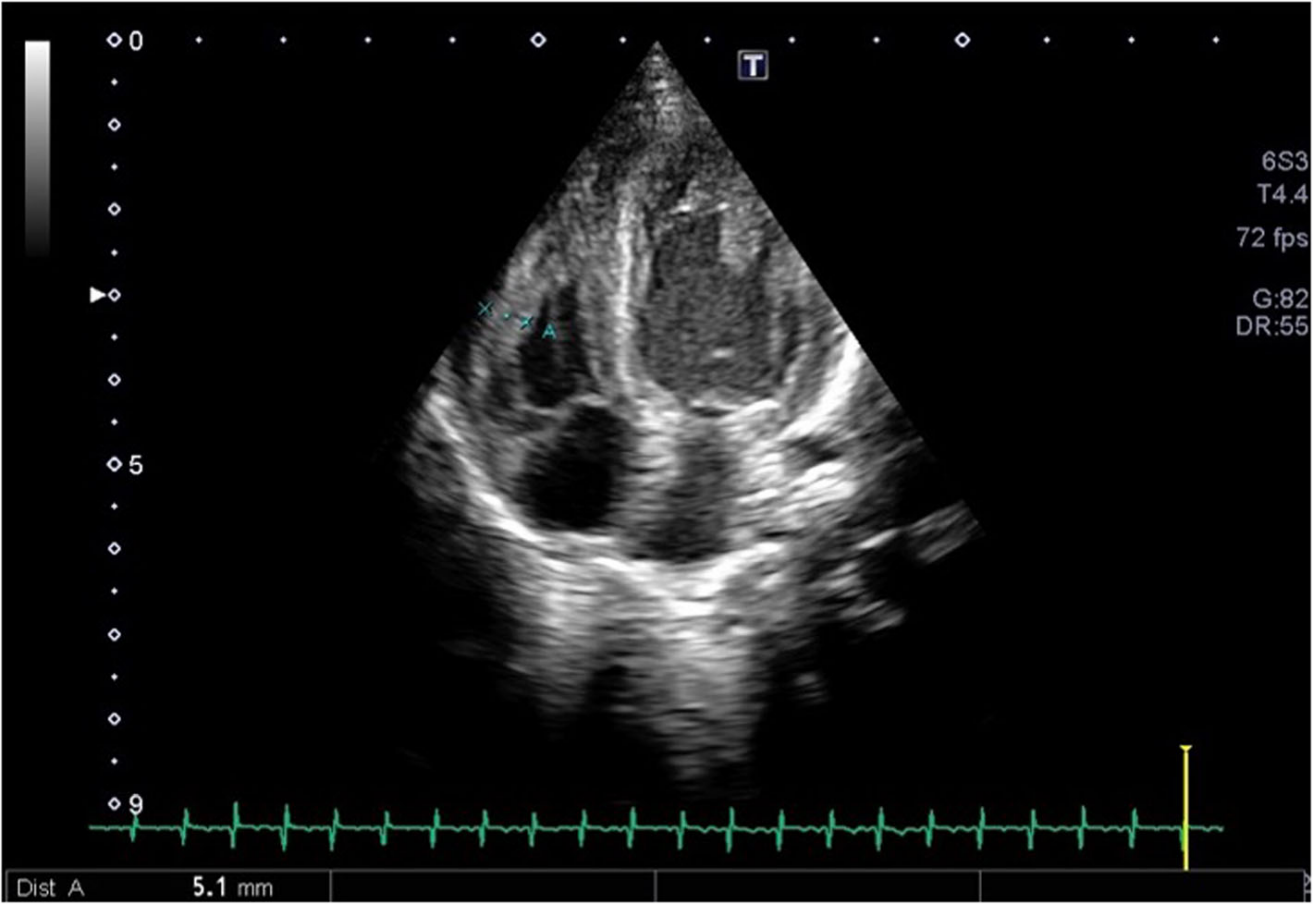
Echocardiogram performed (3 months of age) showed an interventricular septal hypertrophy (SIVd 11.2 mm; z-score 5.1) and a significant reduction in left ventricular cavity (EDV 5 ml) with ecocardiographic signs of LV outflow tract obstruction (maximum intraventricular pressure gradient 7.9 mmHg)

The last echocardiogram performed (3 months of age) showed an interventricular septal hypertrophy (SIVd 11.2 mm; z-score 5.1) and a significant reduction in left ventricular cavity (EDV 5 ml) with ecocardiographic signs of LV outflow tract obstruction (maximum intraventricular pressure gradient 7.9 mmHg) (Fig. 4). During hospitalization, K.A. was feeded by nutrient-enriched formula milk with a better weight gain and improving general conditions. Finally, she has been transferred to a reference center for Pompe patients in order to start Enzyme Replacement Therapy (ERT), currently ongoing.

## Discussion

We report two clinical cases of neonates, born to mothers respectively with a pre-pregnancy insulin-dependent diabetic mellitus type 2 and a suspected diabetes, with inadequate prenatal glycemic control for the first and underestimated glycemic control for the second case. Clinical and echocardiographic findings for each case revealed a severe hypertrophic cardiomyopathy and were similar at the presentation. The low weight of the second case was attributed to both twin pregnancy and possible placental diabetic microangiopathy.

Pregestational diabetes is well known to be a significant risk factor for congenital heart anomalies, such as Ventricular and Atrial Septal defects (VSD, ASD), Patent Ductus Arteriosus (PDA), Transposition of Great vessels (TGA), Coarctation of Aorta (CoA) [7]. HCM is often observed in infants of diabetic mothers (IDMs), with an incidence varying between 10 and 71% [8]. Fetal outcome in IDMs is usually related to the period of onset in the mother and duration of maternal glucose intolerance during pregnancy besides the severity of the mother’s diabetes [4]. Fetuses of mothers with type 1 diabetes have the highest risk of developing HCM, followed by type 2 diabetes and only a low percentage in gestational diabetes [4, 5].

The hypertrophic cardiomyopathy observed in fetuses exposed to hyperglycemic and hyperinsulinemic conditions during diabetic pregnancies are primarily affecting the interventricular septum because of the large number of insulin receptors in the septum of the heart, but can extend to the myocardium in more severe cases [8]. Septal hypertrophy is found by echocardiography in 30% of all IDMs and can reproducibly be detected as early as the 18th week of pregnancy, whereas progressive thickening can be documented up through at least the 33rd week of gestation [9]. Although septal hypertrophy is less common in well-controlled pregnant diabetic women, it remains uncertain whether producing tighter glucose control after the diagnosis of fetal ventricular septal hypertrophy will lead to regression of the hypertrophy and reduce the severity of this condition.

However, it is important to remember that neonatal HCM is an heterogenous condition which can subtend several disorders, potentially difficult to distinguish if only based on the first cardiac ultrasound approach.

In children, HCM may be progressive and serial ECG and echocardiographic measurements of LV dimension, wall thickness ad degree of obstruction, can conduct diagnostic program.

Our cases represent two examples of HCM in newborns of diabetic mother in which the follow-up has been crucial to identify the underlying diagnosis: in the first case, during the cardiological follow-up a slow evidence of improvement of the HCM was observed, persuading us to the diagnosis of a diabetes-related HCM; in the second case the progressive worsening of the HCM during follow-up in association with very typical ECG findings lead us to perform further investigations, resulted in the diagnosis of a metabolic glycogen storage disorder: Pompe disease. The prognosis of HCM depends on the underlying diagnosis: HCM related to maternal diabetes generally resolves without complications within 6 months after birth, eventually with appropriate therapy (eg, propranolol or other beta blockers). In Pompe disease patients usually show a progressive cardiac HCM and rarely survive beyond one year of age, with cardiac failure as main cause of death. In early onset Pompe disease infants are often floppy at birth. Since the introduction of ERT the survival has increased significantly due to reduced cardiac hypertrophy and improved cardiac function; however, little is known about ERT’s long term effects on the heart [10].

## Conclusions

Hypertrophic cardiomyopathy in newborns can be the clinical expression of different underlying disorders. In infants of diabetic mothers HCM is commonly found, particularly if there is a poorly controlled prenatal glycemia. All IDMs with septal hypertrophy should receive consultation and follow-up with a pediatric cardiologist, working in collaboration with the gynecologist: fetal monitoring, delivery planning and childbirth care are required.

After birth, it is important both to reassess maternal and family history and critically evaluate the physical examination in order to address the correct differential diagnosis. Furthermore it is important to continue a regular cardiologic follow-up with ECG, echocardiography and Holter ECG when indicated, in order to evaluate the progression of the HCM: generally HCM in IDMs has a good prognosis and this cardiomyopathy is usually reversible. When HCM doesn’t improve or worsens further investigations in order to exclude metabolic disorders are promptly needed, as these are characterized by poor prognosis in the first year of life.

## Data Availability

The datasets used and analyzed during the current study are available from. the corresponding author on reasonable request.

## Abbreviations

HCM: Hypertrophic cardiomyopathy
IDMs: Infants of Diabetic Mother
SAM: Systolic anterior motion
SIVd: Interventricular septal hypertrophy diameter
EDV: End Diastolic Volume
ASD: Atrial Septal defects
PDA: Patent Ductus Arteriosus
TGA: Transposition of Great vessels
CoA: Coarctation of Aorta
DBS: Dried Blood Spot
ERT: Enzyme Replacement Therapy
LV: Left Ventricular
